# Comparing effects of CDK inhibition and E2F1/2 ablation on neuronal cell death pathways in vitro and after traumatic brain injury

**DOI:** 10.1038/s41419-018-1156-y

**Published:** 2018-11-06

**Authors:** Taryn G. Aubrecht, Alan I. Faden, Boris Sabirzhanov, Ethan P. Glaser, Brian A. Roelofs, Brian M. Polster, Oleg Makarevich, Bogdan A. Stoica

**Affiliations:** Department of Anesthesiology and Shock, Trauma and Anesthesiology Research (STAR) Center, University of Maryland School of Medicine, Baltimore, MD USA

## Abstract

Traumatic brain injury (TBI) activates multiple neuronal cell death mechanisms, leading to post-traumatic neuronal loss and neurological deficits. TBI-induced cell cycle activation (CCA) in post-mitotic neurons causes regulated cell death involving cyclin-dependent kinase (CDK) activation and initiation of an E2F transcription factor-mediated pro-apoptotic program. Here we examine the mechanisms of CCA-dependent neuronal apoptosis in primary neurons in vitro and in mice exposed to controlled cortical impact (CCI). In contrast to our prior work demonstrating robust neuroprotective effects by CDK inhibitors after TBI, examination of neuronal apoptotic mechanisms in E2F1^−/−^/E2F2^−/−^ or E2F2^−/−^ transgenic mice following CCI suggests that E2F1 and/or E2F2 likely play only a modest role in neuronal cell loss after brain trauma. To elucidate more critical CCA molecular pathways involved in post-traumatic neuronal cell death, we investigated the neuroprotective effects and mechanisms of the potent CDK inhibitor CR8 in a DNA damage model of cell death in primary cortical neurons. CR8 treatment significantly reduced caspase activation and cleavage of caspase substrates, attenuating neuronal cell death. CR8 neuroprotective effects appeared to reflect inhibition of multiple pathways converging on the mitochondrion, including injury-induced elevation of pro-apoptotic Bcl-2 homology region 3 (BH3)-only proteins Puma and Noxa, thereby attenuating mitochondrial permeabilization and release of cytochrome c and AIF, with reduction of both caspase-dependent and -independent apoptosis. CR8 administration also limited injury-induced deficits in mitochondrial respiration. These neuroprotective effects may be explained by CR8-mediated inhibition of key upstream injury responses, including attenuation of c-Jun phosphorylation/activation as well as inhibition of p53 transactivation of BH3-only targets.

## Introduction

Traumatic brain injury (TBI) is a major public health problem, with 2.8 million cases reported in 2013^1^. Approximately 50 000 people die annually from TBI, and survivors often suffer from significant and life-long neurological disabilities^1,2^. TBI leads not only to direct and immediate brain damage, primary injury, but also initiates delayed and progressive molecular and cellular changes, secondary injury, which contribute substantially to the overall neuronal loss and ultimately to the post-traumatic cognitive, motor, and affective neurological dysfunctions^3,4^. The secondary injury processes, including various neuronal cell death mechanisms, are initiated at the lesion site as early as the first minutes after trauma but may persist for much longer at sites distant from the core lesion and represent key targets for therapeutic interventions^5–7^. We and others have shown that TBI triggers a chronically progressive neurodegeneration and tissue loss, which is associated with functional impairments and highlighted the importance of identifying the responsible neuronal cell death pathways^8–11^.

Previous studies have indicated that activation of the cell cycle machinery in post-mitotic neurons results in ce`ll death and have shown the importance of cell cycle activation (CCA) as a secondary injury mechanism after TBI^12–14^. CCA leads to apoptosis in post-mitotic cells such as neurons and oligodendroglia and may also contribute to microglia proliferation, neuroinflammation, and secondary neurotoxicity^15,16^. Inhibition of the cell cycle with cyclin-dependent kinase (CDK) inhibitors attenuates neuronal apoptosis in vitro and provides neuroprotection after TBI^9,17–20^. CDKs phosphorylate retinoblastoma (Rb) family proteins, promoting dissociation of the Rb-E2F complexes followed by activation of the E2F transcription factors and upregulation of E2F-dependent genes^21^. E2F1, -2, and -3 may promote cell death by inducing the expression of pro-apoptotic molecules such as caspases, apoptotic protease-activating factor 1 (Apaf-1), or Bcl-2 homology region 3 (BH3)-only proteins^22,23^. Rb phosphorylation and E2F1 activation following experimental TBI in rats may play a role in post-traumatic neuronal apoptosis^24^. Furthermore, the role of E2F1 in neuronal apoptosis is suggested by studies showing that cortical neuronal cultures and hippocampal slices from E2F1^−/−^ knockout mice are protected against neurotoxicity following oxygen and glucose deprivation compared to E2F1^+/+^ mice^25^.

Our previous studies have shown that inhibition of the E2F1/CDK1 pathway attenuates neuronal apoptosis in vitro and is neuroprotective after spinal cord injury (SCI)^26,27^. The goal of the current study was to determine the role of E2F1/2 transcription factors in neuronal loss after controlled cortical impact (CCI), a well-established mouse experimental TBI model, and to characterize the neuroprotective mechanisms of CDK inhibitors in a DNA damage model of neuronal injury in vitro.

## Results

### E2F2^−/−^ and E2F1^−/−^/E2F2^−/−^ mice show reduced neuronal loss in the dentate gyrus after TBI but no attenuation of acute injury markers or gene expression changes in the hippocampus

**Fig. 1:**
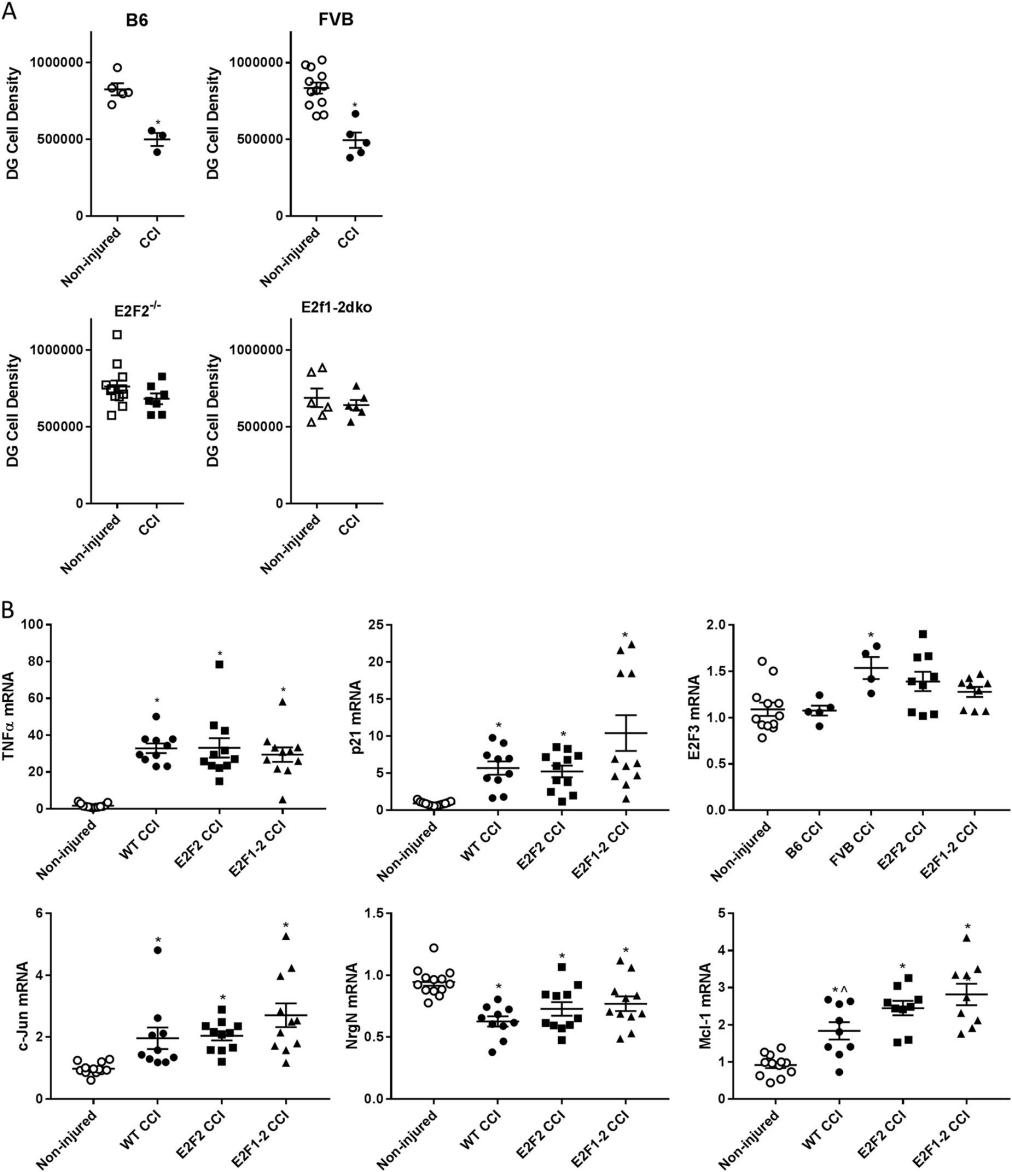
E2F2^−/−^ and E2F1^−/−^/E2F2^−/−^ mice show reduced neuronal loss in the dentate gyrus (DG) after TBI but no attenuation of acute injury markers or gene expression changes in the hippocampus. Cell count stereology data for the DG of the hippocampus at 28 days post injury (**a**). B6 non-injured *n* = 5, CCI *n* = 3; FVB non-injured *n* = 12 (6 ipsilateral and 6 contralateral), CCI *n* = 5; E2F2^−/−^ non-injured *n* = 12 (6 ipsilateral and 6 contralateral), CCI *n* = 7; E2F1^−/−^/E2F2^−^^/−^ non-injured *n* = 6 (3 ipsilateral and 3 contralateral), CCI *n* = 6. Data represent mean ± SEM, * indicates significant difference from non-injured control via two-tailed *t*-test. CCI increased gene expression of TNF, p21, Jun, and Mcl-1 in the hippocampus regardless of genotype. CCI decreased gene expression NrgN in the hippocampus regardless of genotype. Only FVB CCI mice had increased E2F3 gene expression (**b**). B6 non-injured *n* = 4 (2 ipsilateral and 2 contralateral), CCI *n* = 5; FVB non-injured *n* = 3, CCI *n* = 5; E2F2^−/−^ non-injured *n* = 4, CCI *n* = 11; E2F1^−/−^/E2F2^−/−^ non-injured *n* = 4, CCI *n* = 11. Non-injured controls include non-injured mice from all the genotypes, WT CCI group combines both wild-type backgrounds (B6 and FVB). Data represent mean ± SEM of one-way ANOVA and Tukey post hoc analysis, *p < 0.05 vs. non-injured control

CCI reduced neuronal density in the dentate gyrus (DG) in B6129SF2/J (*T*(6) = 5.31, *p* = 0.001) and FVB mice (*T*(15) = 5.28, *p* = 0.001) compared to their non-injured counterparts. We observed no reduction in DG neuronal density in injured vs. non-injured E2F2^−/−^ (*T*(17) = 1.38, *p* = 0.1868) or E2F1^−/−^/E2F2^−/−^ mice (*T*(10) = 0.693, *p* = 0.5038) (Fig. 1a).

We observed an injury-induced increase in *tumor necrosis factor alpha (TNFα)* (*H*(3) = 27.63, *p* = 0.0001), *cyclin-dependent kinase inhibitor 1A-CDK1A (p21Cip/WAF1)* (*H*(3) = 27.31, *p* = 0.0001),* c-Jun* (*H*(3) = 26.49, *p* = 0.0001), and* myeloid cell leukemia sequence 1 (Mcl-1)* (*F*(3,35) = 18.86, *p* = 0.0001) mRNA levels in each genotype. *E2F3* (*F*(3,34) = 4.287, *p* = 0.0065) was unchanged with injury except for FVB mice. *Neurogranin (NrgN)* (*F*(3,41) = 8.37, *p* = 0.0002) expression was downregulated after CCI in all genotypes compared to non-injured mice. For all tested mRNAs no differences were observed in wild-type (FVB and B6129SF2/J) vs. E2F2^−/−^ and wild-type vs. E2F1^−/−^/E2F2^−/−^ mice after CCI (*p* > 0.05); for Mcl-1 no differences were observed in E2F2^−/−^ vs. wild-type but E2F1^−/−^/E2F2^−/−^ was higher than wild-type after CCI (*p* > 0.05) (Fig. 1b).

### E2F2^−/−^ and E2F1^−/−^/E2F2^−/−^ mice show no attenuation in acute injury markers in cortex

**Fig. 2:**
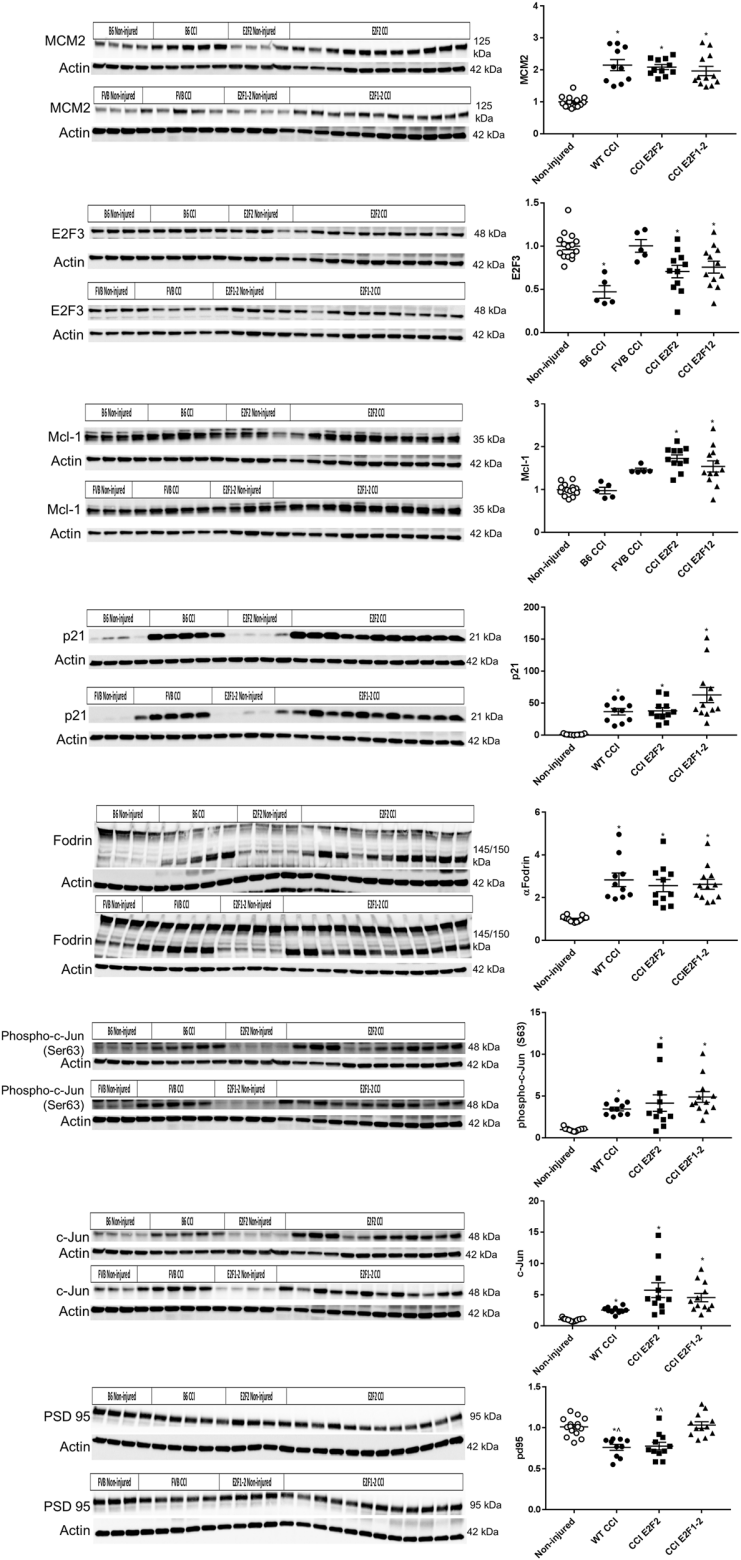
E2F2^−/−^ and E2F1^−/−^/E2F2^−/−^ mice show no attenuation in acute injury markers at the protein level in cortex. Whole-tissue lysates from non-injured mouse cortices and 24 h after TBI were fractioned on SDS-polyacrylamide gel and immunobloted with antibodies against MCM2, E2F3, Mcl-1, p21, a-Fodrin, phospho-c-Jun (Ser63), c-Jun, and PSD95. Protein levels were quantified by densitometry, normalized to β-actin, and presented as fold change compared with non-injured mice. Non-injured controls include all the non-injured mice from all the genotypes, CCI group combines the B6 and FVB genotypes (WT CCI). B6 non-injured *n* = 4, CCI *n* = 5; FVB non-injured *n* = 3, CCI *n* = 5; E2F2^−/−^ non-injured *n* = 4, CCI *n* = 11; E2F1^−/−^/E2F2^−/−^ non-injured *n* = 4, CCI *n* = 12. Data represent mean ± SEM of one-way ANOVA and Tukey post hoc analysis, *p < 0.05 vs. non-injured control, ^*p* < 0.05 vs. E2F1^−/−^/E2F2^−/−^. For MCM2, Mcl-1, c-Jun, phospho-c-Jun, α-Fodrin, p21, and Jun data represent mean ± SEM of Kruskal-Wallis test with Dunn’s multiple comparisons post hoc test, *p < 0.05 vs. non-injured control, ^*p* < 0.05 vs. E2F1^−/−^/E2F2^−/−^

We observed an injury-induced elevation in minichromosome maintenance complex component 2 (MCM2) (*H*(3) = 31.01, *p* = 0.0001), p21 (*H*(3) = 18.46, *p* = 0.0052), cleaved α-Fodrin (spectrin) p145/150 fragments (*H*(3) = 30.56, *p* = 0.0001), phospho-c-Jun (Ser63) (*H*(3) = 29.06, *p* = 0.0001), and c-Jun (total) (*H*(3) = 34.72, *p* = 0.0001) in each genotype following CCI compared to non-injured mice. We observed an injury-induced elevation in Mcl-1 (*H*(4) = 29.38, *p* = 0.0001) in E2F2^−/−^ and E2F1^−/−^/E2F2^−/−^ but not wild type compared to non-injured control. We observed an injury-induced decrease in E2F3 (*F*(4,43) = 8.84, *p* = 0.0001) except for the FVB CCI group. For MCM2, p21, cleaved α-Fodrin p145/150, phospho-c-Jun (Ser63), c-Jun, E2F3, and Mcl-1 no protein level differences were observed in wild-type vs. E2F2^−/−^ and wild-type vs. E2F1^−/−^/E2F2^−/−^ mice after CCI (*p* > 0.05), except E2F2^−/−^ being increased compared to B6129SF2/J for Mcl-1. A reduction in post-synaptic density protein 95 (PSD95) levels (*F*(3,44) = 14.95, *p* = 0.0001) was induced following CCI in wild-type mice (WT CCI) and E2F2^−/−^ compared to non-injured mice and E2F1^−/−^/E2F2^−/−^ mice (*p* < 0.01) (Fig. 2).

### CR8 decreases etoposide-induced cell death in primary rat cortical neurons

**Fig. 3:**
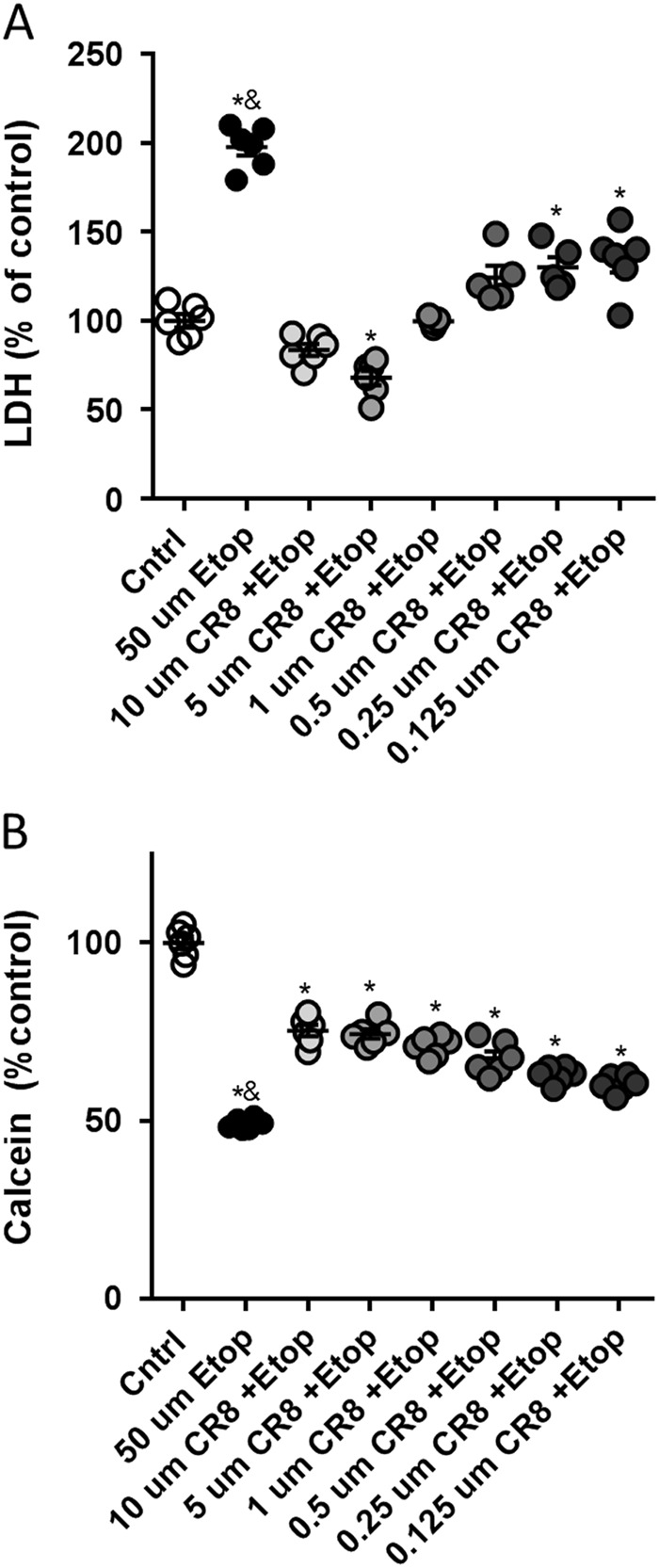
CR8 decreases etoposide-induced cell death in primary neurons. Neurons were treated with 50 μm of etoposide ± 10, 5, 1, 0.5, 0.25, and 0.125 μm CR8. Cell death, LDH release (**a**), and cell viability, Calcein signal (**b**), were measured 24 h after treatment. Histograms indicates LDH release and Calcein signal as percentage of control untreated RCN. *n* = 6/group for all groups. Data represent mean ± SEM of one-way ANOVA and Tukey post hoc analysis, *p < 0.05 vs. control, ^&^*p* < 0.05 vs. etoposide + CR8

Etoposide-treated primary rat cortical neurons (RCN) showed increased lactate dehydrogenase (LDH) release (*F*(7,38) = 59.06, *p* = 0.0001) compared to control (*p* < 0.05) at 24 h after treatment (Fig. 3a). Etoposide + CR8 (at all concentrations of CR8) attenuated LDH release compared to etoposide (*p* < 0.05). Etoposide-treated RCN showed decreased calcein signal (*F*(7,40) = 122.4, *p* = 0.0001) compared to control RCN (*p* < 0.05) at 24 h (Fig. 3b). Etoposide + CR8 (at all concentrations of CR8) demonstrated increased Calcein signal compared to etoposide (*p* < 0.05) (Fig. 3b).

### CR8 attenuates activation of the p53-dependent pro-apoptotic pathways following DNA damage

**Fig. 4:**
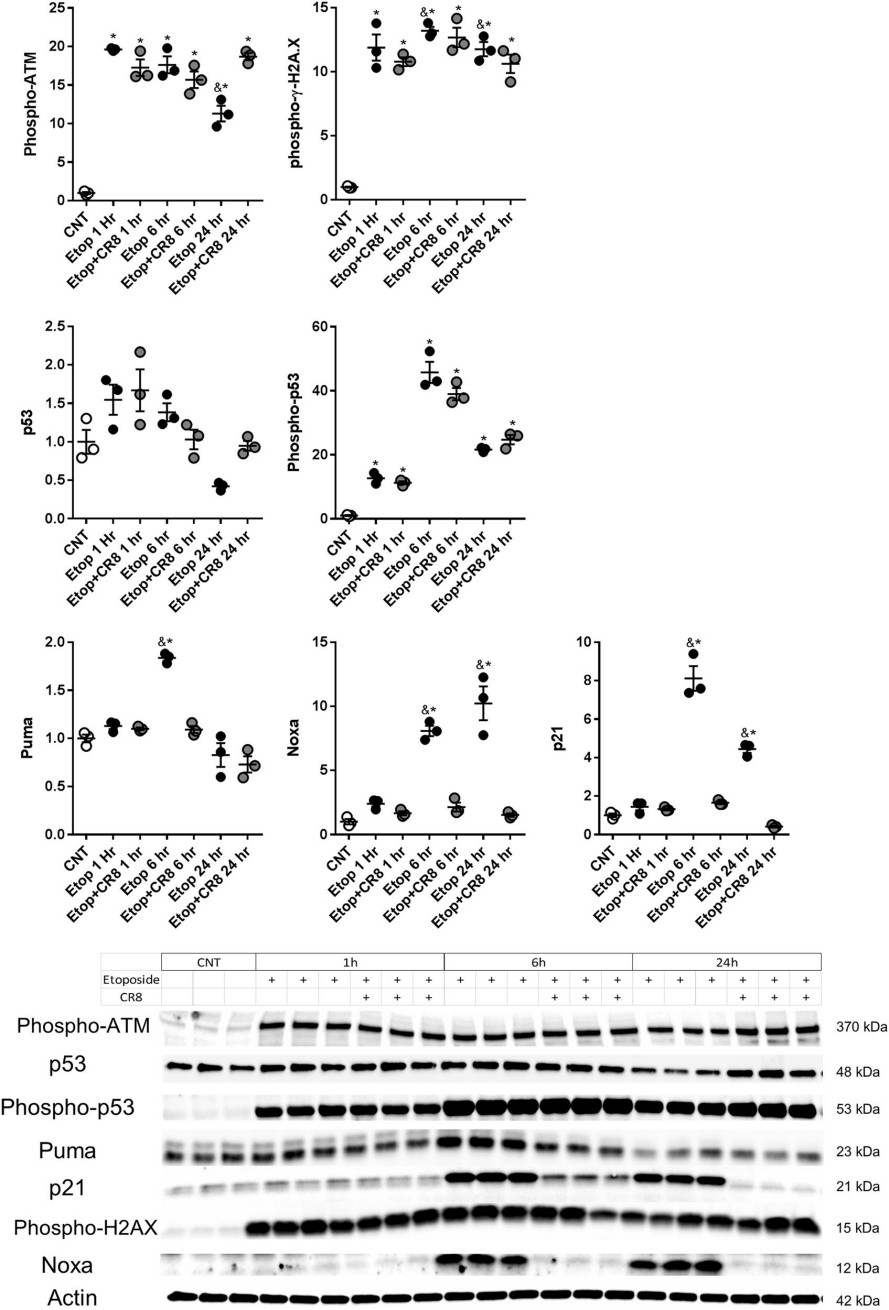
CR8 attenuates activation of the p53-linked pro-apoptotic pathways following etoposide-induced DNA damage. Neurons were treated with 50 μm of etoposide ± 1 μm CR8. Twenty-four hours later whole-cell lysates were fractioned on SDS-polyacrylamide gel and immunoblotted with antibodies against phospho-ATM, γ-H2A.X, p53, phospho-p53, Puma, Noxa, and p21. Protein levels were quantified by densitometry, normalized to β-actin, and presented as fold change compared with control untreated levels. Cell death occurs in all neurons treated with 50 μm etoposide (phospho-ATM and γ-H2A.X) including an increase in phospho-p53. However, 1 μm CR8 attenuates the etoposide-induced increase in downstream targets of p53 (Puma, Noxa, and p21). *n* = 3/group for all groups. Data represent mean ± SEM of one-way ANOVA and Tukey post hoc analysis, *p < 0.05 vs. control, ^&^*p* < 0.05 vs. etoposide + CR8 at the same time point

We observed an increase in phosphorylated ataxia telangiectasia mutated kinase (phospho-ATM) (*F*(6,14) = 62.29, *p* = 0.0001) and γ-H2A.X protein levels (*F*(6,14) = 113, *p* = 0.0001) in etoposide and etoposide + CR8 RCN compared to control (*p* < 0.0001) (Fig. 4). Phospho-ATM levels were similar in etoposide and etoposide + CR8, although at 24 h etoposide + CR8 was higher than etoposide (*p* < 0.001). γ-H2A.X were similar in etoposide and etoposide + CR8 although at both 6 and 24 h etoposide + CR8 decreased compared to etoposide (*p* < 0.05). Total p53 protein levels were not different among groups (*p* > 0.05). Phospho-p53 protein (*F*(6,14) = 96.82, *p* = 0.0001) was increased in etoposide- and etoposide + CR8-treated RCN at all time points compared to control (*p* < 0.01). Phospho-p53 levels were not different in etoposide and etoposide + CR8 treatment (*p* > 0.05). Etoposide increased p53-upregulated modulator of apoptosis (Puma) (*F*(6,14) = 32.66, *p* = 0.0001), phorbol-12-myristate-13-acetate-induced protein 1 (Noxa) (*F*(6,14) = 44.12, *p* = 0.0001), and p21 (*F*(6,14) = 104.1, *p* = 0.0001) levels compared to controls (*p* < 0.0001). Puma, Noxa, and p21 levels were lower in etoposide + CR8 compared to etoposide (*p* < 0.0001) (Fig. 4).

**Fig. 5:**
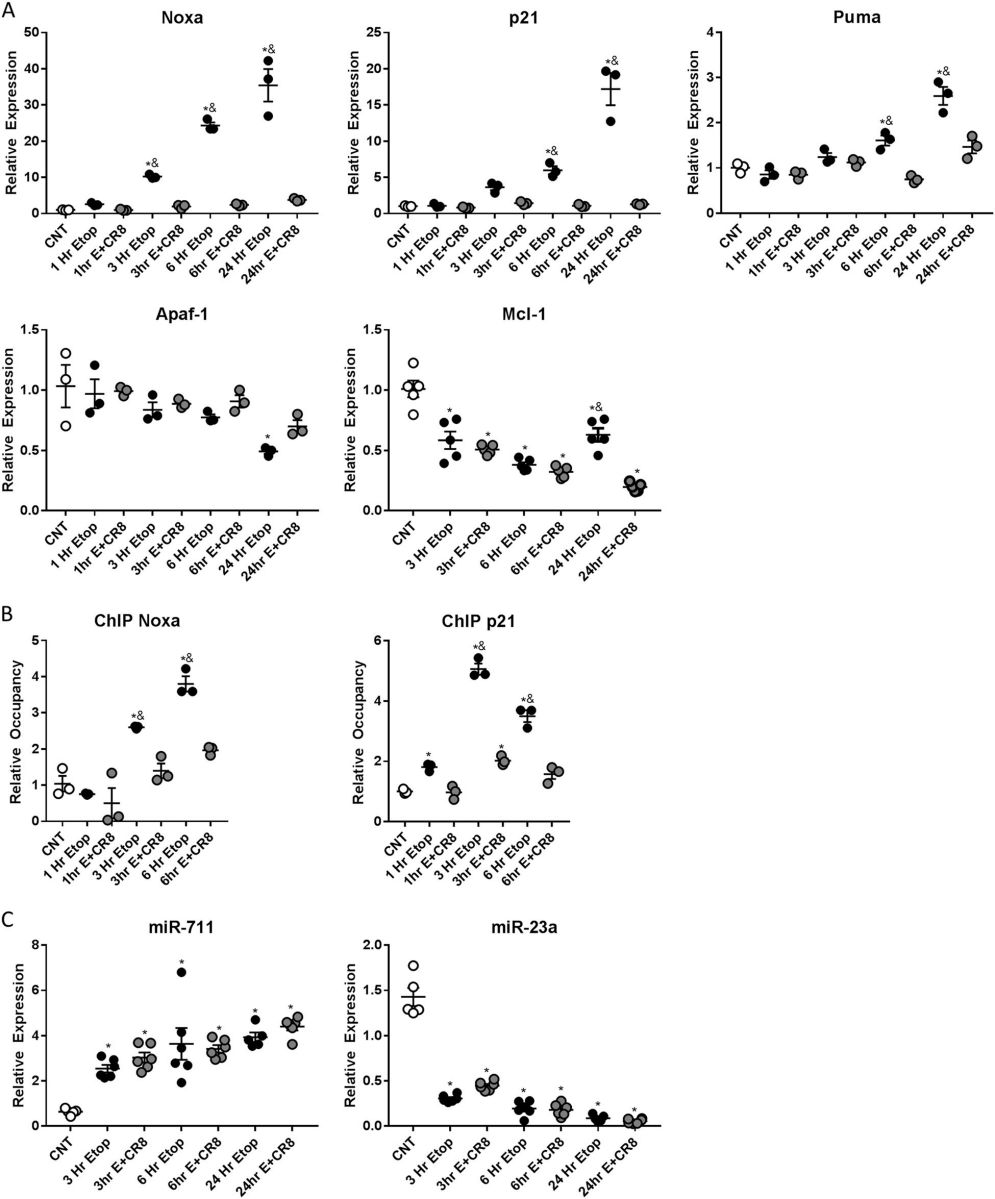
CR8 attenuates activation of the p53-linked pro-apoptotic pathways following etoposide-induced DNA damage at the mRNA level. Neurons were treated with 50 μm of etoposide ± 1 μm CR8. Neurons were collected 24 h after treatment. qPCR quantification of expression of **a** Noxa, p21, Puma, Apaf-1, and Mcl-1; **b** promotor region of Noxa and p21; **c** miR-711 and miR-23a in primary cortical neurons at different time points after treatment. Results of qPCR were normalized to **a** GAPDH expression; **b** input DNA; and **c** U6 snRNA. CR8 attenuates relative expression of PUMA, NOXA, and p21 following 50 μm etoposide treatment (**a**). No change in Apaf-1 relative to controls was observed until 24 h (**a**). Etoposide induced increases in occupancy of p53 in the promoter region of Noxa, and p21 was attenuated by etoposide + CR8 (**b**). *n* = 3/group for all groups. Etoposide and etoposide + CR8 increased expression of miR-711 and decreased miR-23a compared to control neurons (**c**). *n* = 6/group for all groups. Data represent mean ± SEM of one-way ANOVA and Tukey post hoc analysis, *p < 0.05 vs. control, ^&^*p* < 0.05 vs. etoposide + CR8 at the same time point

*NOXA*(*F*(8,18) = 64.45, *p* = 0.0001), *p21* (*F*(8,18) = 46.55, *p* = 0.0001), and *PUMA* (*F*(8,18) = 29.57, *p* = 0.0001) expression was increased in etoposide compared to control. *NOXA*, *p21*, and *PUMA* expression was attenuated in etoposide + CR8 treatment compared to etoposide (*p* < 0.01). *Apaf-1* (*F*(8,18) = 4.541, *p* = 0.0037) levels were unchanged among groups (*p* > 0.05), except decreased levels in etoposide compared to control at 24 h (*p* < 0.005). *Mcl-1* (*F*(6,28) = 33.45, *p* = 0.0001) expression was unchanged between etoposide and etoposide + CR8 except at 24 h when etoposide + CR8 was lower than etoposide (*p* < 0.01) (Fig. 5a). p53 occupancy on the promotor region of NOXA (*F*(6,14) = 30.47, *p* = 0.0001) and p21 (*F*(6,14) = 118.4, *p* = 0.0001) increased in etoposide compared to control (*p* < 0.01); and was attenuated in etoposide + CR8 at 3 and 6 h compared to etoposide (*p* < 0.01) (Fig. 5b). Etoposide and etoposide + CR8 increased *miR-711*(*F*(6,33) = 13.36, *p* = 0.0001) and decreased *miR-23a* (*F*(6,33) = 135.3, *p* = 0.0001) expression relative to controls at all time points (*p* > 0.005). Etoposide-treated neurons had similar levels of *miR-711* and *miR-23a* as etoposide + CR8 (*p* > 0.05) (Fig. 5c).

### CR8 reduces etoposide-induced activation of the c-Jun injury response pathway

**Fig. 6:**
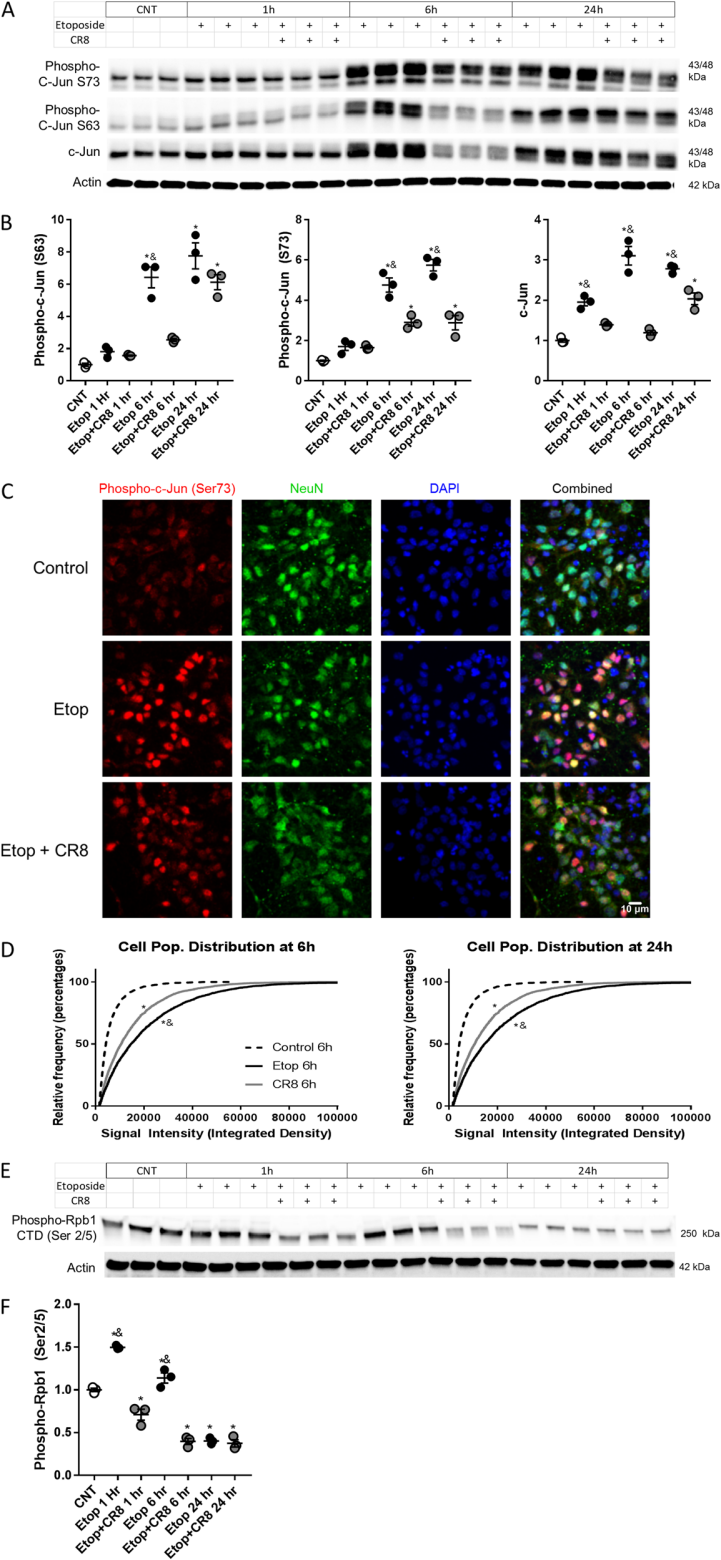
CR8 reduces etoposide-induced activation of the c-Jun injury response pathway in primary neurons. **a**, **b**, **e**, **f** Neurons were treated with 50 μm of etoposide ± 1 μm CR8. Twenty-four hours later whole-cell lysates were fractioned on SDS-polyacrylamide gel and immunoblotted with antibodies against phospho-c-Jun, c-Jun, and phospho-Rpb1 CTD (Ser2/5). Protein levels were quantified by densitometry, normalized to β-actin, and presented as fold change compared with control untreated levels. **c** Neurons were fixed 24 h after treatment with formaldehyde and stained with antibodies for phospho-c-Jun (Ser73), NeuN, and DAPI for fluorescent imaging. CR8 attenuates 50 μm etoposide-induced increases in phospho (Ser63 and 73) and total c-Jun expression compared to etoposide treatment alone, *n* = 3/group for all groups (**a**). Data represent mean ± SEM of one-way ANOVA and Tukey post hoc analysis, *p < 0.05 vs. control, ^&^*p* < 0.05 vs. etoposide + CR8 at the same time point. Representative image from 6 h confocal microscopy of coverslips stained for NeuN (green), p-c-Jun (red), and DAPI (blue) (**c**). Data were calculated and plotted for all fields together as a cumulative frequency distribution of fluorescent intensity without binning (**d**). Kruskal-Wallis test followed by Dunn’s post hoc analysis, all groups had significantly different distributions; *p < 0.05 vs. control, ^&^*p* < 0.05 vs. etoposide + CR8 (*p* < 0.05). Etoposide-induced increases in phospho-Rpb1 CTD (Ser2/5) are attenuated in CR8 treated neurons at early time points. Western images (**e**) graphed in **f**, *n* = 3/group for all groups. Data represent mean ± SEM of one-way ANOVA and Tukey post hoc analysis, *p < 0.05 vs. control, ^&^*p* < 0.05 vs. etoposide + CR8 at the same time point

Phospho-c-Jun (Ser63) (*F*(6,14) = 40.01, *p* = 0.0001) (6 and 24 h), phospho-c-Jun (Ser73) (*F*(6,14) = 51.93, *p* = 0.0001) (6 and 24 h), and c-Jun (total) (*F*(6,14) = 48.76, *p* = 0.0001) (1, 6, and 24 h) levels increased after etoposide treatment compared to control neurons (*p* < 0.01). Etoposide-induced increase of phospho-c-Jun (Ser63) (6 h), phospho-c-Jun (Ser73) (6 and 24 h), and c-Jun (1, 6, and 24 h) was attenuated by etoposide + CR8 (*p* < 0.01) (Fig. 6a, b). Phospho-c-Jun (73) staining was more intense at 6 h (*H*(3) = 1956, *p* = 0.0001) and 24 h (*H*(3) = 894.2, *p* = 0.0001) in etoposide compared to control (*p* < 0.05). Etoposide + CR8 attenuated phospho-c-Jun (73) intensity compared to etoposide (*p* < 0.05) (Fig. 6c, d). Phospho-Rpb1 CTD (Ser2/5) (RNA polymerase II) protein levels (*F*(6,14) = 116, *p* = 0.0001) were increased with etoposide (1 and 6 h) compared to control. Etoposide + CR8 attenuated phospho-Rpb1 CTD (Ser2/5) levels (1 and 6 h) compared to etoposide (*p* < 0.001) (Fig. 6e, f).

### CR8 attenuates etoposide-induced mitochondrial dysfunction and permeabilization in primary cortical neurons

**Fig. 7:**
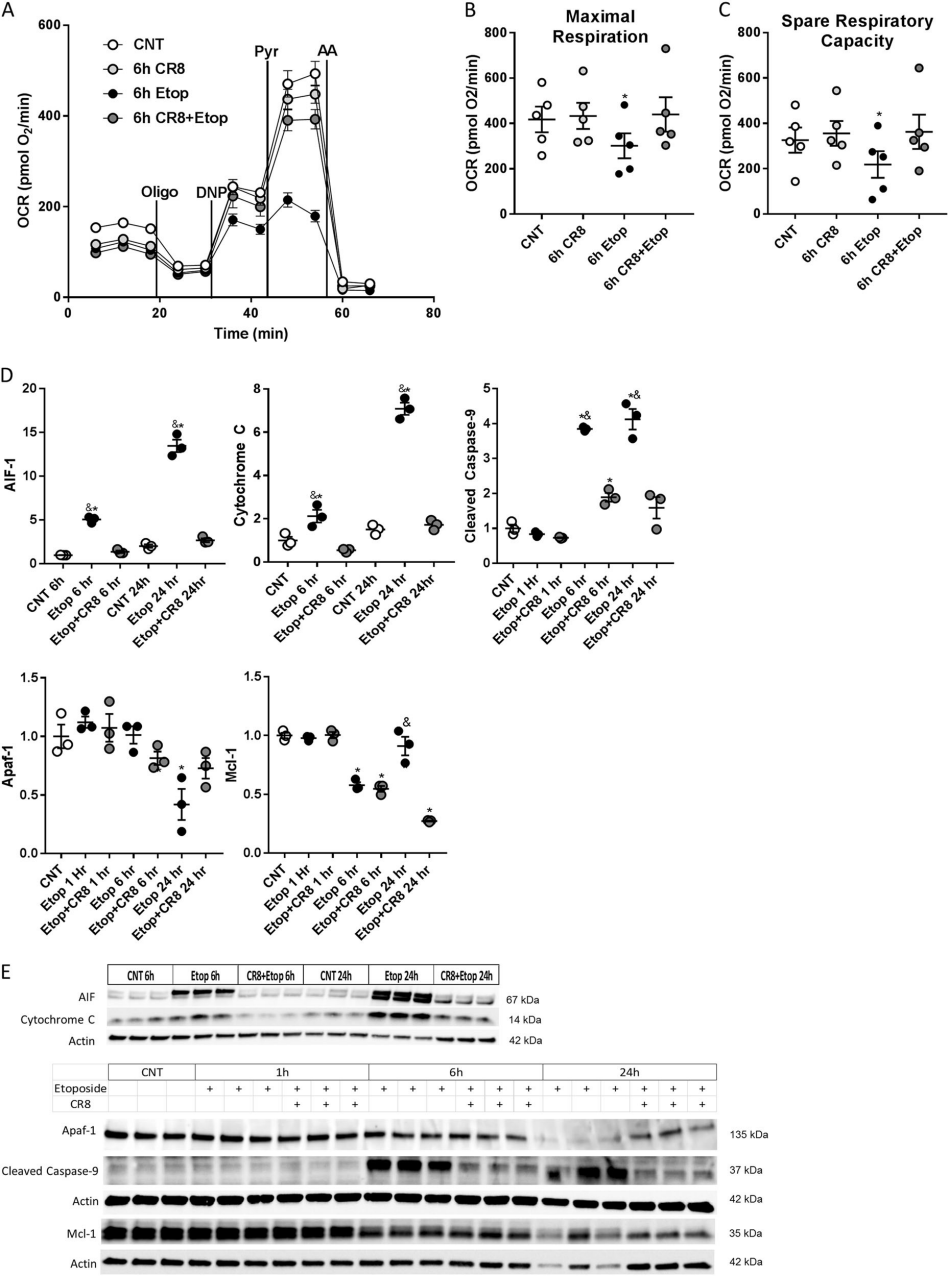
CR8 attenuates etoposide-induced mitochondrial dysfunction and mitochondrial permeabilization in primary neurons. Neurons were treated with 50 μM of etoposide ± 1 μM CR8. Fractions and whole lysates were collected 24 h later and proteins were separated on SDS-polyacrylamide gels, then immunodetected using antibodies against AIF-1, cytochrome C, cleaved caspase-9, Apaf-1, and Mcl-1. Protein levels were quantified by densitometry, normalized to β-actin, and presented as fold change compared with control untreated levels. In parallel, 6 h after treatment cellular respiration measurements were made with a Seahorse XF24 Extracellular Flux Analyzer before and after sequential additions of oligomycin, FCCP, pyruvate, and antimycin A at the indicated times (representative experiment (**a**)). CR8 rescues maximal respiration (**b**) and spare respiratory capacity (**c**) compared to etoposide treatment alone. *n* = 4 averages from separate days of experiments; on each day, *n* = 4 technical replicates for control, *n* = 5 technical replicates for all other groups. CR8 attenuates the increase in AIF-1 and cytochrome C in the cytosolic fraction and cleavage of caspase-9 in total lysate of rat primary cortical neurons following etoposide treatment (**d**). Western images (**e**) quantified in **d**. No change in Apaf-1 relative to controls was observed until 24 h. Both etoposide and etoposide + CR8 decreased Mcl-1 at 6 h, at 24 h etoposide alone increased relative to etoposide + CR8 (**d**). *n* = 3/group for all groups for westerns. Data represent mean ± SEM of one-way ANOVA and Tukey post hoc analysis, *p < 0.05 vs. control, ^&^*p* < 0.05 vs. etoposide + CR8 at the same time point

Maximal respiration (*F*(2.12,8.47) = 8.76, *p* = 0.0082) and spare respiratory capacity (*F*(2.19,8.75) = 10.46, *p* = 0.0043) were lower in etoposide compared to controls or CR8 treatment alone (*p* < 0.05). Maximal respiration and spare respiratory capacity were higher in etoposide + CR8 compared to etoposide (*p* < 0.05) (Fig. 7a, b). At 6 h after etoposide treatment no increase in LDH or decrease in calcein was observed (*p* > 0.05, data not shown).

The level of cytosolic apoptosis-inducing factor mitochondria associated 1 (AIF-1) (*F*(5,12) = 202.2, *p* = 0.0001), cytosolic cytochrome C (*F*(5,12) = 149.5, *p* = 0.0001), and cleaved caspase-9 (*F*(6,14) = 66.38, *p* = 0.0001) increased in etoposide compared to control (*p* < 0.05). The levels of cytosolic AIF-1, cytosolic cytochrome c, and caspase-9 were reduced in etoposide + CR8 compared to etoposide (*p* < 0.05). Apaf-1 levels in control, etoposide, and etoposide + CR8 were similar (*p* > 0.05), except for the 24 h decrease in etoposide compared to control (*F*(6,14) = 7.098, *p* = 0.0013). Mcl-1 (*F*(6,14) = 65.5, *p* = 0.0001) was decreased in etoposide at 6 h compared to control (*p* < 0.001). Mcl-1 expression was unchanged between etoposide and etoposide + CR8 except at 24 h when etoposide + CR8 was lower than etoposide (*p* < 0.001) (Fig. 7d, e).

### CR8 reduces etoposide-induced caspase activation in primary cortical neurons

**Fig. 8:**
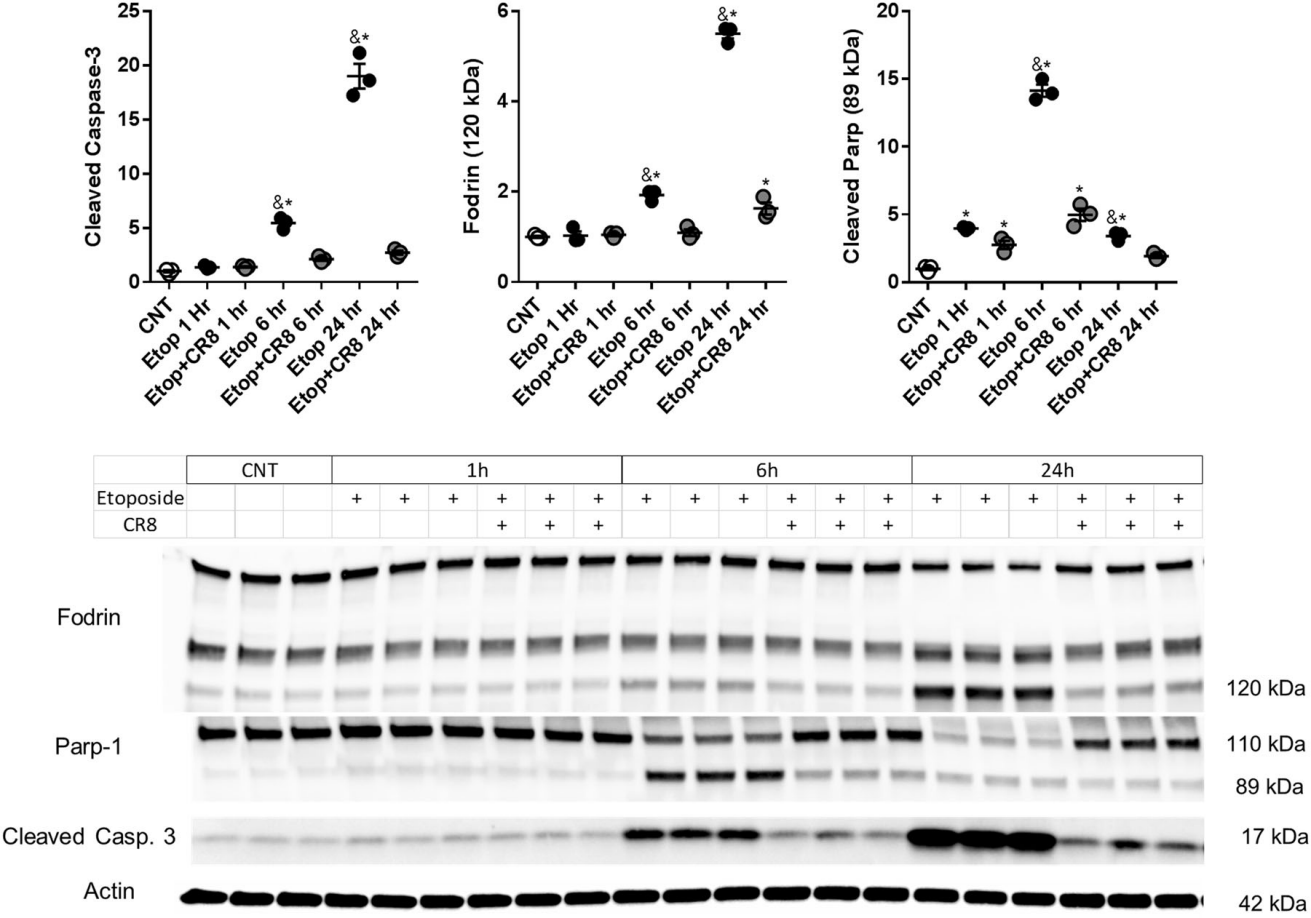
CR8 reduces etoposide-induced caspase activation in primary neurons. Neurons were treated with 50 μm of etoposide ± 1 μm CR8. Twenty-four hours later whole-cell lysates were fractioned on SDS-polyacrylamide gel and immunoblotted with antibodies against cleaved caspase-3, -αFodrin, and -Parp. Protein levels were quantified by densitometry, normalized to β-actin, and presented as fold change compared with control untreated levels. *n* = 3/group for all groups. Data represent mean ± SEM, * indicates significant difference from control, ^&^ indicates significant difference from CR8-treated neurons at the same time point, *p* < 0.05, via one-way ANOVA and Tukey post hoc test (**b**)

Etoposide increased cleavage of caspase-3 (*F*(6,14) = 194.4, *p* = 0.0001), α-Fodrin (120 kDa) (*F*(6,14) = 379.6, *p* = 0.0001), and poly (ADP-ribose) polymerase (PARP) (89 kDa) (*F*(6,14) = 239.5, *p* = 0.0001) compared to controls (*p* < 0.03). Etoposide-induced increase of cleaved caspase-3, -α-Fodrin, and -PARP were attenuated in etoposide + CR8 (*p* < 0.03) (Fig. 8).

## Discussion

In response to various insults, post-mitotic neurons undergo CCA that triggers apoptotic pathways and leads to neuronal cell death^12^. The specific cell cycle mechanisms and downstream cell death cascades involved in neuronal injury have yet to be delineated. A major goal of this study was to examine the role of E2F1 and E2F2 in neuronal loss following mouse CCI, an experimental TBI model. E2F2^−/−^ single-knockout and E2F1^−^^/−^/E2F2^−/−^ double-knockout transgenic mice showed no injury-induced chronic neuronal loss in the DG, a key region of the hippocampus at 28 days post TBI. In contrast, wild-type mice had a CCI-induced decrease in neuronal cell density. To examine the mechanisms responsible for the neuroprotective effects of E2F2 and E2F1/2 ablation, we measured TBI-induced transcriptional activation of various acute injury response genes in hippocampus after brain trauma including: TNFα, whose rapid increase in neurons after TBI contributes to subsequent neurological dysfunction^28^; CDKI1A-p21^CIP1/WAF1^, a molecule induced by the apoptotic master regulator p53 during neuronal cell death^29,30^; and c-Jun, a member of the AP-1 transcription factor family and an important mediator of stress-induced neuronal apoptosis^31–33^. Our data indicate that neither E2F2 nor E2F1/2 ablation attenuates the increased hippocampal gene expression of TNFα, p21, c-Jun, and Mcl-1 at 24 h after trauma compared to wild-type controls. Furthermore, E2F2 and E2F1/2 ablation did not reduce acute TBI-induced hippocampal downregulation of NrgN, a marker of synaptic degeneration and neuronal damage after brain trauma^34^.

We also examined the acute changes in several key cell cycle and cell death proteins in the injured cortex. TBI-induced elevations of MCM2 (a marker of CCA in neurodegeneration^35^), c-Jun (total), or p21 were not attenuated by E2F2 and E2F1/2 ablation. The transcriptional activity of c-Jun is activated by injury-induced phosphorylation of Ser63/Ser73, located in the NH2-terminal transactivation domain, and leads to expression of pro-apoptotic genes and neuronal apoptosis^36^. The TBI-induced increase in c-Jun pSer63 levels was not attenuated in E2F2^−/−^ or E2F1^−/−^/E2F2^−/−^. TBI-induced cleavage of Fodrin, a specific marker of neuronal cell death after TBI^34^, was not attenuated in E2F2^−/−^ or E2F1^−/−^/E2F2^−/−^, whereas downregulation of PSD95, a marker of synaptic degeneration and neuronal damage^37,38^ following injury, was reduced by E2F1/2 but not by E2F2 ablation.

Overall, our in vivo data show no attenuation of neuronal injury markers in E2F2^−/−^ or E2F1^−/−^/E2F2^−/−^ transgenics compared to wild-type controls, except for PSD95 with E2F1/2 ablation, suggesting that E2F1/2 may play only a minor role in acute neuronal cell death after TBI. In contrast, our experimental SCI studies have shown robust attenuation of early neuronal apoptosis in E2F1^−/−^/E2F2^−/−^ transgenics^27^. Furthermore, studies of experimental models of focal cerebral ischemia have also described that the absence of E2F1 has neuroprotective effects^39^. These studies likely reflect pathobiological differences in acute neuronal responses in TBI as compared to SCI or ischemia. Our in vitro studies also indirectly suggest that E2F1 may not play a broad role in neuronal cell death. Thus, we observed no upregulation in the mRNA or protein levels of Apaf-1, a key target of E2F1-mediated apoptosis^40^ in RCN treated with etoposide, an inducer of DNA damage-mediated neuronal cell death. The observed reduction of chronic neuronal loss in the hippocampus after TBI in E2F2^−/−^ or E2F1^−/−^/E2F2^−/−^ may therefore reflect attenuation of microglial or astroglial activation, as was shown by our previous SCI studies^27^.

The inability of even the dual E2F1/2 knockout to inhibit neuronal cell death mechanisms may reflect functional redundancy among the large E2F family^41^; independent roles of E2F1^42^, E2F2^43^, and E2F3^23^ in apoptosis; the relative downstream position of E2Fs in cell cycle pathways; or perhaps their narrower role involving only selected neuronal cell death models. E2F3 is another member of the activating E2F family with pro-apoptotic activity^23,44^. No differences in E2F3 levels in wild-type vs. E2F2^−/−^ or E2F1^−/−^/E2F2^−/−^ mice were observed after CCI, suggesting the absence of an E2F3 compensatory upregulation. Nonetheless, we cannot exclude the possibility that E2F3 may contribute to the post-traumatic neuronal cell death in the E2F1/2 knockout animals. To better examine the CCA-dependent neuronal apoptosis pathways after injury, we therefore targeted more upstream mechanisms using the potent CDK inhibitor CR8^45^. Previously, we showed that CR8 reduced post-traumatic neuronal cell loss and neuroinflammation, attenuating neurological deficits in mouse (CCI) and rat (lateral fluid percussion) TBI models^19,46^. Activation of p53, followed by induction of BH3-only pro-apoptotic molecules and mitochondrial permeabilization, plays a critical role in acute neuronal death after injury^47,49^. Key elements of these pathways are present in a model of etoposide-induced DNA double-strand breaks (DSBs) in primary cortical neurons to trigger p53-BH3-dependent apoptosis^34,50^. Dose-response studies using two independent measurements of neuronal loss showed that concurrent CR8 administration at a dose as low as 1 μM robustly attenuates etoposide-induced neuronal cell death. Following etoposide-induced DSBs, neurons activate the ATM/p53 apoptosis pathways that include sequential phosphorylation of ATM on Ser1981 and phosphorylation of histone H2A.X on Ser128 (γH2A.X). ATM-mediated p53 Ser15 phosphorylation is critical for transactivation of pro-apoptotic BH3-only molecules such as Puma and Noxa^51^ through increased binding to coactivator proteins such as p300 and/or DNA^52–54^. CR8 had no consistent effect on etoposide-induced pSer1981ATM and γH2A.X elevation, suggesting that it may act downstream of the induction of DSBs and ATM activation.

The activation of p53 in response to etoposide reflects substantial increases in p53 Ser15 phosphorylation, without changes in total p53. Although we observed no CR8 effects on the levels of either of Ser15 phosphorylated p53 or total p53, we showed robust CR8-dependent inhibition of etoposide-induced regulation of p53 targets such as the pro-apoptotic molecules Puma, Noxa, and p21 at both the mRNA and protein levels. CDK5 can induce neuronal apoptosis by phosphorylating/activating ATM^55^ and/or p53^56^. Although CR8 is a potent inhibitor of many CDKs, including CDK5^45^, we did not observe any changes in etoposide-induced ATM phosphorylation, suggesting that CDK5 does not play a key role in our model. Instead, as indicated by our chromatin immunoprecipitation (ChIP) studies, CR8 may inhibit p53 activity by reducing etoposide-induced p53 localization at the promoter of pro-apoptotic molecules such as Noxa or p21. The mechanisms responsible for the CR8-dependent p53 regulation may involve c-Jun, a molecule previously found to be required for the function of other members of the pro-apoptotic p53 family^57^ and that can be phosphorylated and activated by CDKs^58,59^. Inhibition of c-Jun following treatment with CDK inhibitors is associated with attenuation of neuronal apoptosis in sympathetic neurons exposed to DNA-damaging agents^60^. Furthermore, c-Jun and p53 pathways may be activated independently and interact cooperatively to trigger the expression of BH3-only molecules in neuronal apoptosis^61^. Overall, these observations are consistent with our data, demonstrating that CR8 administration strongly attenuates etoposide-induced elevation in total and Ser63/73 phosphorylated c-Jun. Positive binding cooperativity between diverse transcription factors and CBP/p300, coactivators known to regulate crosstalk between various cellular signaling pathways^62^, may underpin the p53/c-Jun interactions. Other key apoptotic mechanisms converging on the BH3-only molecules and mitochondria, including apoptosis-associated decrease of miR-23a^47^ or elevation of miR-711^50^, were not affected by CR8.

Mcl-1 is an anti-apoptotic Bcl-2 family member that has been shown to be rapidly downregulated after DNA damage in neurons and maintaining high Mcl-1 levels can protect neurons against death^63^. CDKs such as CDK5 phosphorylate Mcl-1 inducing its degradation followed by mitochondria dysfunction and death in a model of glutamate-induced neurotoxicity in primary neurons; Mcl-1 degradation and neurotoxicity can be attenuated by CDK inhibitors^64^. In contrast to their neuroprotective effect in specific post-mitotic neuron models, CDK inhibitors may have opposite effects in neuroblastoma and other proliferating neuronal cell lines where they cause downregulation of Mcl-1 and lead to apoptosis^65^. In our model, Mcl-1 was rapidly downregulated in the first 6 h followed by partial recovery at 24 h after etoposide treatment. Administration of CR8 did not change the Mcl-1 response profile until 24 h, at which time its levels where lower than with etoposide alone, suggesting CR8 may lower Mcl-1 levels following neuronal DNA damage. E2F1 may directly repress the expression of Mcl-1^66^. Our data indicate that the injury-induced Mcl-1 increase may be more robust following E2F1/2 ablation (especially E2F1^−/−^/E2F2^−/−^) compared to wild-type control. Ablation of E2F1/2 may promote higher Mcl-1 expression, an effect that could contribute to the observed attenuation in neuronal loss.

An alternative explanation for the CR8-mediated downregulation of pro-apoptotic molecules may involve the inhibition of CDK9, a regulator of the RNA transcription machinery. CR8, which is a potent inhibitor of CDK9^45^, can attenuate CDK9-dependent phosphorylation/activation of RNA polymerase II^45^ and may reduce protein synthesis^60^. Our data are, in part, consistent with these findings and show that CR8 decreases the etoposide-induced phosphorylation of Rpb1 C-terminal domain, the largest subunit of RNA polymerase II. Nonetheless, our data are not consistent with ﻿global repression of gene transcription.

A key element in all types of regulated cell death is the poorly defined “point-of-no-return,” which in etoposide-induced neuronal cell death may reflect irreversible and widespread mitochondrial outer membrane permeabilization (MOMP)^67^. CR8 neuroprotective effectiveness may be due to its ability to inhibit pathways upstream and converging upon mitochondria; these may include mechanisms leading to BH3-only molecules, which, unlike post-mitochondrial interventions, may prevent and not just delay cell death^67^. In our model, the “point-of-no-return” may be reached by 6 h, when increasing activation of upstream pathways leads to MOMP with release of pro-apoptotic mitochondrial molecules cytochrome c and AIF-1 into the cytosol to initiate intrinsic caspase-dependent and -independent apoptosis, respectively. Importantly, we detected no late markers of neuronal cell death at this time point. CR8 administration not only attenuates the release of cytochrome c and AIF-1 but also inhibits the later steps in the final execution phase of the apoptosis cascade^67^. This includes activation of caspase-9 and caspase-3, as well as cleavage of caspase substrates such as Fodrin and PARP-1. CR8 also preserves mitochondrial bioenergetic function after etoposide treatment.

In summary, our in vivo study highlights the limitations of constitutive genetic targeting of E2F1/2 as a neuroprotective intervention in TBI. This may be due to the downstream position of E2F1/2 in the signaling cascades and their potentially restricted role limited to specific cell death paradigms. It may reflect limitations of the constitutive knockout of E2Fs, resulting in synaptic disruptions, neurogenesis reductions, and behavioral deficits^68^. Future studies that use cell-specific and inducible gene ablation may provide a better assessment of the specific role of E2Fs in TBI. CR8 attenuation of neuronal cell death was associated with inhibition of key apoptotic mechanisms, such as BH3-only proteins upstream of the mitochondria. Moreover, CR8-mediated neuroprotection occurs downstream of p53 phosphorylation and may involve inhibition of c-Jun phosphorylation/activation and its cooperative interaction with p53. Overall, these results demonstrate the ability of CR8 to target key neuronal cell death mechanisms, such as the mitochondrial apoptotic pathway, and establish CDK inhibition as a promising neuroprotective intervention.

## Materials and methods

### Mice

Breeding pairs of male and female transgenic E2F1^+/−^E2F2^−/−^ mice were received from Dr. Gustavo Leone (The Ohio State University). The breeding was carried out at the University of Maryland School of Medicine Laboratory Animal Resource Center. Male E2F1^−/−^ E2F2^−/−^ (E2F1-2 double-knockout) or E2F2^−/−^ (E2F2 single-knockout) mice >8 weeks old (approximately 18–25 g) used in this study were generated from 10 breeding pairs. A total of 25 male E2F1^−/−^ E2F2^−/−^ and 28 male E2F2^−/−^ were used in this study. Nineteen 8-week-old (20–25 g) male FVB mice were obtained from JAX (Jackson laboratories, Bar Harbor, ME). Fifteen 8-week-old (20–25 g) male B6129SF2/J were obtained from JAX (Jackson laboratories, Bar Harbor, ME). As E2F mice have a mixed genetic background there is no ideal control strain; FVB and B6129SF2/J are both part of the E2F knockout background and both were used as controls as previous studies have shown significant strain differences in regard to neurological responses following CCI^69^. Mice were maintained on a 12-h light/dark cycle with ad libitum access to food and water. All activities were in accordance with protocols approved by the University of Maryland School of Animal Care and Use Committee and complied with the Guide for the Care and Use of Laboratory Animals published by NIH (DHEW publication NIH 85-23-2985).

### Experimental TBI model: CCI

We used a custom-designed CCI-injury device^9,70^ consisting of a microprocessor, controlled with a pneumatic impactor that has a 3.5-mm diameter tip. Mice were anesthetized with isoflurane (induced at 3% and maintained at 1.5%) in a 70% nitrous oxide, 30% oxygen gas mixture, administered through a nose mask. Anesthesia depth was assessed by monitoring respiration rate and pedal withdrawal reflexes. The surgical site was clipped then the head was mounted in a stereotaxic frame and the site was cleaned with betadine (Professional Disposables, Orangeburgy, NY) and ethanol scrubs (Fisher Scientific, Hampton, NH). Mice received puralube vet ointment eye lubrication (Dechra Veterinary Products, Overland Park, KS). Then a 10-mm midline incision was made over the skull, the skin and fascia were reflected, and a 5-mm craniotomy was made on the central aspect of the left parietal bone^46^. The impounder tip of the injury device was extended to its full stroke distance (44 mm) and positioned to the surface of the exposed dura then reset to impact the cortical surface. Moderate injury was achieved using an impactor velocity of 6 m/s and a deformation depth of 2 mm, as previously described^46^. Following impact, the incision was closed with 9 mm wound clips (Stoelting Co., Wood Dale, IL), and anesthesia was then terminated. Next, mice were placed into a chamber with heated and non-heated sections and monitored continuously until sternal recumbency was regained and then intermittently for 45 min. All animals were monitored for at least 4 h after injury and then daily. Sham mice underwent the same procedure as injured mice except no impact occurred. Mice were randomly assigned to CCI or sham group.

### Histology

Twenty-eight days after injury a subset of mice were anesthetized (100 mg/kg sodium pentobarbital intraperitoneally), transcardially perfused with ice-cold saline and 4% paraformaldehyde. Order of euthanasia was randomly alternated between CCI and sham groups and genotypes. Brains were post-fixed in paraformaldehyde for 24 h then transferred to 20% sucrose for ~24 h until brains sunk and then were transferred to 30% sucrose until embedding. Frozen brain sections (60 and 20 μm) were cut on a cryostat and mounted onto glass slides. Selected slides were stained with cresyl violet for unbiased assessment of neuronal cell loss in the hippocampus.

### Stereological assessment of neuronal cell loss in the hippocampus

Steroinvestigator software (MBF Biosciencies, Williston, VT) was used to count the total number of surviving neurons in the cornu ammonis DG of the hippocampus using the optical fractionator method of unbiased stereology. Every fourth 60 μm section between −1.22 and −2.54 mm from bregma was analyzed, beginning from section 1, 2, or 3 randomly across mice samples (5 sections were assessed per mouse). The optical dissection had a size of 50 μm by 50 μm in the *x*- and *y*-axis, respectively, with a height of 10 μm and a guard-zone of 4 μm from the top of the section. The sampled region for each hippocampal subfield was demarcated in the injured hemisphere and cresyl violet neuronal cell bodies were counted. For the DG, a grid spacing of 175 μm in the *x*-axis and 100 μm in the *y*-axis was used, resulting in an area fraction of one-twenty-eighth. The volume of the traced hippocampal subfield was provided in the program output. The estimated number of surviving neurons in each field was divided by the volume of the region of interest to obtain the neuronal cellular density, expressed as counts/mm^3^, as previously described^46^. Cutting, histology, and cell counting was performed by a treatment and genotype blinded individual, though CCI brains are typically distinguishable by eye from non-injured mice once sectioned.

### Primary cortical neuronal cultures

RCN were derived from rat embryonic cortices. Cells were seeded onto poly-d-lysine-coated 96 well, 12-well, 60 mm, or 100 mm Petri dishes, or XF24 cell culture microplates (Agilent, Santa Clara, CA) (cell density 1 × 10^6^/cm^2^) and maintained in serum-free conditions using the B27 supplement as described previously^47^. Etoposide is an inhibitor of DNA topoisomerase II, causing DNA breaks and caspase-3-dependent apoptosis. At 7 days in vitro (DIV) cells were treated with etoposide (Enzo Life Sciences, Farmingdale, NY) at a final concentration of 50 μM for 1, 3, 6, or 24 h at which point assays were performed or cells collected. Replicates for in vitro experiments are from the same RCN but different wells or plates.

### Mitochondrial respiration measurements

Cellular oxygen consumption was measured using the Seahorse XF24 Extracellular Flux Analyzer (Agilent, Santa Clara, CA) 6 h post-50 μM etoposide treatment of DIV 7 primary RCN plated at cell density 1 × 10^6^/cm^2^ cells/well. Thirty minutes before etoposide treatment some cells received a 1 μM CR8 pretreatment in conditioned media. Experiments were performed in artificial cerebral spinal fluid consisting of 120 mM NaCl, 3.5 mM KCl, 1.3 mM CaCl_2_, 0.4 mM KH_2_PO_4_, 1 mM MgCl_2_, 4 mg/ml fatty acid-free bovine serum albumin, 5 mM 4-(2-hydroxyethyl)-1-piperazineethanesulfonic acid (HEPES), and 15 mM glucose (pH 7.4) at 37 °C^48^. Final concentrations of injected drugs were 0.5 μg/ml oligomycin, 200 μM dinitrophenol, 10 mM pyruvate (Pyr), and 1 μM antimycin A (AA). Pyr was added to ensure endogenous substrate supply was not rate limiting during the measurement of maximal respiration. Addition of the electron transport chain complex III inhibitor AA confirmed that cellular oxygen consumption was almost exclusively of mitochondrial origin. The results consist of four experiments; each data point represents the average of five internal repeats.

### Neuronal cell death and cell viability assays

Cell death and cell viability were measured as previously described using LDH (G1780 Promega, Madison, WI) and Calcein AM (ALX-610-026-m001 Enzo Life Sciences, Farmingdale, NY) assays, respectively^71^ on BioTek Synergy Ht Microplate Reader (BioTek, Winooski, VT). Each individual treatment/time point reflect six replicates for all assays performed on cortical neurons cultured in 96-well plates; all wells were plated with and contained the same number of cells.

### RNA isolation and quantitative PCR

Total RNA was isolated using the miRNeasy Kit (QIAGEN, Hilden, Germany) according to the manufacturer’s protocol. Verso cDNA Kit (Thermo Scientific, Waltham, MA) was used to synthesize cDNA from purified total RNA. RNA (1 μg) was heated to 70 °C for 5 min and mixed with 5× cDNA-synthesis buffer, dNTP mix (0.5 nM final concentration), and Verso Enzyme Mix, and finally random hexamers (400 ng/μl) were added. Tubes were incubated at 42 °C for 30 min, followed by 95 °C for 2 min. Quantitative real-time PCR amplification was performed by using cDNA TaqMan Universal Master Mix II (Applied Biosystems, Foster City, CA). Briefly, reactions were performed in duplicate containing 10 µl of 2× TaqMan Universal Master Mix II, 2 μl of cDNA (corresponding to 50 ng RNA/reaction), and 1 µl of 20× TaqMan Gene Expression assay primers in a final volume of 20 μl. TaqMan Gene Expression assays from Applied Biosystems (Foster City, CA) for the following genes were performed for mouse: *CDKN1a* (p21) (Mm04205640_g1); *TNF* (mm00443258 m1); *Jun* (Mm00495062_s1); *NrgN* (Mm01178296_g1); *E2F3* (Mm01138833_m1); and *Mcl-1* (Mm01257351_g1). TaqMan Gene Expression assays from Applied Biosystems (Foster City, CA) for the following genes were performed for rat: *PUMA* (Rn00597992_m1), *NOXA* (Rn01494552_m1), *CDKN1a* (p21) (Rn00589996_m1), *Apaf-1* (Rn00576832_m1), and *Mcl-1* (Rn00821024_g1). Reactions were amplified and quantified using a 7900HT Fast Real-Time PCR System and the corresponding software (Applied Biosystems, Foster City, CA). The PCR profile consisted of one cycle at 50 °C for 2 min and 95 °C for 10 min, followed by 40 cycles of 95 °C for 15 s and 60 °C for 1 min. Efficiency of reactions was measured using the C_T_ slope method. Gene expression was normalized to GAPDH, and the relative quantity of mRNAs was calculated based on the comparative C_T_ method as previously described^47^.

### miR reverse transcription

Quantitative real-time PCR was used to measure the expression of mature miR-711. A unit of 100 ng of total RNA was reverse-transcribed using miScript II RT Kit (Qiagen). Reverse transcription reaction products (1 µl) were used for quantitative PCR (qPCR) with miScript SYBR Green PCR Kit (Qiagen) according to the manufacturer’s instructions. miScript Primer Assays for following miRs were used:* rno-miR-711* (MS00017696); *rno-miR-23a-3p* (MS00033327); and *U6 snRNA* (MS00033740) (Qiagen).

### ChIP assay

ChIP assays were performed by EpiQuik™ Chromatin Immunoprecipitation (IP) Kit (Epigentek) according to the manufacturer’s instructions. Briefly, 5 × 10^6^ RCN were crosslinked with 10 ml of phosphate-buffered saline (PBS) containing 1% formaldehyde (final concentration). For ChIP 0.5 × 10^6^ of crosslinked RCN were used. Chromatin was sheared to fragments ranging from 200 to 600 bp by Bioruptor sonication device (Diagenode). Immunoprecipitation was performed for 90 min with 1.6 µg of p53 antibodies (Cell Signaling Technology #32532).

Immunoprecipitated DNA and input DNA were analyzed by qPCR with SsoAdvanced™ Universal SYBR^®^ Green Supermix (Bio-Rad). To obtain the fold change in p53 occupancy, data were analyzed using the 2−ΔΔCT method (Livak and Schmittgen, 2001 PMID: 11846609). Results of qPCR were normalized to input (genomic DNA) and gene desert region (nonspecific binding): ΔCT = (Ct of Immunoprecipitation (IP) sample − Ct of input − Ct of gene desert of IP sample). Data were expressed relative to control.

One of predicted p53-binding site in promoter region of rat *p21* gene is located at 7 376 134 nt on chromosome 20 (NC_005119.2). Following primers were designed to amplify this region: p21 forward primer 5′-GGGTACCTGCATGGCTTCTT-3′ (7 376 006–7 376 025 nt); *p21* reverse 5′-CTCCATTCATGCCCCTCCTC-3′ (7 376 265–7 376 246 nt). Predicted p53-binding site in promoter region of rat *Noxa* gene is located at 62 914 518 nt on chromosome 18 (NC_005117.4). These primers were used to amplify this region *Noxa* forward primer 5′-CCACTGTCCCAGCGATGAAC-3′ (62 914 368–62 914 387 nt), *Noxa* reverse 5′-GGCTCTCGGGTTTTATGGGG-3′ (62 914 663–62 914 644 nt). Rat negative control primer set1 (Active Motif) were used to amplify the fragment of a gene desert on rat chromosome 3.

Normal Rabbit IgG (Cell Signaling Technology #32532, #2729) and anti-RNA Polymerase II (Diagenode) antibodies were used as the IP-negative control and positive controls respectively to validate immunoprecipitation procedure. Rat *GAPDH* primers (Epigentek) were used for qPCR with IP-positive and IP-negative control. Level of GAPDH was 57 times lower in IP-negative control compared to IP-positive control (data not shown).

### Cell lysate preparation and western blot

Twenty-four hours after impact a subset of mice were anesthetized (4% isoflurane) and rapidly decapitated. A 5-mm area surrounding the lesion epicenter on the ipsilateral cortex and the hippocampus were rapidly dissected and immediately put on dry ice. For subcellular fractionation, cells were collected with a cell lifter and washed in ice-cold PBS. Cell suspension was centrifuged at 500 × *g* for 15 min at 4 °C as previously described^34^. Cell pellets were resuspended for 10 min on ice in the digitonin lysis buffer (20 mM HEPES, pH 7.4, 80 mM KCl, 1 mM EDTA, 1 mM EGTA, 1 mM dithiothreitol, 250 mM sucrose, 200 μg/ml digitonin, and protease inhibitor and phosphatase inhibitor (2, 3) cocktails (Sigma-Aldrich). The lysate was centrifuged at 1000 × *g* for 5 min at 4 °C to pellet the nuclei. The supernatant was transferred to a new tube and centrifuged again at 12 000 × *g* for 10 min at 4 °C to pellet the mitochondria. The resulting supernatant, representing the cytosolic fraction, was recovered. Nuclear and mitochondrial lysates were prepared in RIPA buffer (Teknova) with Protease Inhibitor Cocktail (Sigma-Aldrich). Protein concentration was determined using Pierce BCA Protein Assay kit (Thermo Scientific, Waltham, MA). Twenty micrograms of protein was run on sodium dodecyl sulfate polyacrylamide gel electrophoresis and transferred onto nitrocellulose membrane. Membranes were washed, and protein complexes were visualized using SuperSignal West Dura Extended Duration Substrate (Thermo Scientific, Waltham, MA). Chemiluminescence was captured on Chemi-Doc Imaging Station (Bio-Rad, Hercules, CA) and protein bands were quantified by densitometric analysis using Image Lab (Bio-Rad, Hercules, CA). Images were acquired under conditions that did not cause saturation of the signal. The data presented reflect the intensity of the target protein band compared with the same genotype control and were normalized based on the intensity of the endogenous control for each sample (expressed in arbitrary units).

### Antibodies

Antibodies from different vendors were used in this study. Antibodies from Cell Signaling (Danvers, MA): MCM2 (3619), Mcl-1 (94296), PSD95 (3450), γ-H2A.X (9718), PARP (9542), cleaved caspase-3 (9661), phospho-p53 (12571), puma (14570), phospho-c-Jun s63 (2361), phospho-c-Jun s73 (9164), c-Jun (9165), ERK1/2 (4695), phospho-ERK1/2 (4370), phospho-Rpb1 CTD (Ser2/5) (4735), and AIF-1 (5318); Enzo (Farmingdale, NY): α-Fodrin (BML-FG6090); Millipore (Ontario, Canada): phospho-Atm (05–740); Abcam (Cambridge, UK): p21 mouse samples (188224) and E2F3 (ab74180); BD Biosciences (San Jose, CA): p21 rat samples (556430); R&D Systems (Minneapolis, MN): p53 (MAB 1355); Sigma-Aldrich (St. Louis, MO): Noxa (PRS2437) and β-actin (A1978); Santa Cruz (Dallas, TX): cytochrome c (sc-7159).

### Immunohistochemistry

For immunocytochemistry, cells were treated with CR8 ± etoposide on DIV 7 in 24-well plates with coverslips. After 6 or 24 h, coverslipped RCN were fixed for 10 min in 4% paraformaldehyde/PBS and then co-stained with a 1:200 dilution of Cell Signaling’s phospho-c-Jun (Ser73) (catalog no: D47G9) antibody and a 1:500 dilution of NeuN (Millipore, Ontario, Canada; catalog no: MAB377) in 10% goat serum (Gemini Bio-Prodcuts, West Sacramento, CA) overnight at 4 °C. Wells were incubated with goat-derived secondary antibody (Life Technologies, Fisher Scientific, Hampton, NH) on the next day and then incubated with 4′,6-diamidino-2-phenylindole (DAPI; Sigma-Aldrich, St. Louis, MO; catalog no: D8417) (0.5 µg/ml in saline) for 30 min.

Imaging was performed via a Leica SP5 II confocal microscope utilizing a ×20 (dry) objective and analysis done using ImageJ software. Optimal settings were determined such that signal intensity was maximized in the 6 h control samples without oversaturating the detector for the 24 h etoposide sample. Settings were maintained for all coverslips to allow for equivalent comparisons of signal intensity.

For each coverslip, four fields were randomly chosen and a series of 1024 × 1024 pixel images was collected at a resolution of 8 bits with a *z*-slice distance of 0.5 µm. Each field was separately and automatically analyzed for phospho-c-Jun fluorescent intensity via FIJI (ImageJ). For each set of images for a given field (*z*-stack), the fluorescent intensity across the *z*-stack was summed to generate a single image (maximum projection). A threshold value of 120 was applied to the signal seen in this stacked image to eliminate background signal. Using the “Analyze Particles” tool on ImageJ, the signal intensity of particles of size 10–150 pixels was calculated. This lower bound was chosen to eliminate additional background signal while the upper bound was chosen as based on the DAPI signal, no single cell was found to be larger than 150 pixels. Each “particle” (cell) was analyzed to determine the total amount of signal within the boundaries of the cell. Each cell’s total signal (integrated density) was graphed as a data point in an unbinned cumulative frequency distribution^72^. This process was applied to all samples.

### Statistical analysis

All statistics were performed using Graphpad Prism 7 (La Jolla, CA). Two-tailed *t*-test was used to analyze DG cell counts. One-way repeated-measures ANOVA was used for mitochondrial function. One-way ANOVAs with Tukey post hoc tests were used to analyze: western blot, qPCR, ChIP, LDH, and calcein assays except for: in vivo MCM2, Mcl-1, c-Jun, phospho-c-Jun, α-Fodrin, TNFα, and p21, which violated the assumption of normality (Shapiro-Wilk test) and were analyzed using a Kruskal-Wallis test with Dunn’s multiple comparisons post hoc test. For in vivo qPCR and western analyses non-injured mice from each genotype (B6; FVB; E2F2^−/−^; and E2F1^−/−^/E2F2^−/−^) were combined into a single non-injured category as a one-way ANOVA showed no statistical differences between any of the non-injured groups. Both wild-type background genotypes, B6129SF2/J and FVB, were combined into a single injury group (WT CCI) when a two-tailed *t*-test indicated no differences between the B6129SF2/J and FVB injured groups. Immunohistochemically stained cells were also analyzed by Kruskal-Wallis test followed by Dunn’s post hoc analysis. In vitro experiments were repeated three times.
